# Isotopic Constraints on SO_2_ Oxidation Rates and Their Potential Relationship with Sulfate Formation Pathways in the Planetary Boundary Layer

**DOI:** 10.1021/acsenvironau.4c00070

**Published:** 2024-12-19

**Authors:** Zhengwen Niu, Mang Lin

**Affiliations:** 1 State Key Laboratory of Isotope Geochemistry, Guangzhou Institute of Geochemistry, Chinese Academy of Sciences, Guangzhou 510640, China; 2 College of Earth and Planetary Sciences, University of Chinese Academy of Sciences, Beijing 100039, China

**Keywords:** radiosulfur, cosmogenic ^35^S radionuclide, triple oxygen isotope, sulfate aerosols, California, Tibetan Plateau

## Abstract

Natural and anthropogenic emissions of sulfur-bearing species significantly alter the sulfur and energy budgets of the Earth’s atmosphere. Simulations of the atmospheric sulfur cycle, sulfate radiative forcing, and predictions of their future changes require a precise understanding of the SO_2_ oxidation rates that control the formation of secondary sulfate aerosols. Given the unique single source of radiosulfur (cosmogenic ^35^S radionuclide), combined measurements of atmospheric radiosulfur in both sulfur dioxide (^35^SO_2_) and sulfate (^35^SO_4_^2–^) have been employed to constrain sulfur oxidation rates in the atmosphere. This approach employed box model calculations, incorporating several key assumed parameters, including sulfur deposition rates. However, previous calculations did not fully consider uncertainties in parametrizations, necessitating a re-examination of the estimated values. In this study, we applied a new approach to revisit existing combined measurements of ^35^SO_2_ and ^35^SO_4_^2–^ at coastal and inland sites. We estimated the temporospatial variability in SO_2_ oxidation rates by incorporating a comprehensive consideration of parametrization uncertainties. We adopted deposition data from nine models of the Atmospheric Chemistry and Climate Model Intercomparison Project. Uncertainties in deposition data and other key parameters, such as cosmogenic ^35^S production rates and ^35^SO_2_/^35^SO_4_^2–^ ratios in the free troposphere, were evaluated by using a Monte Carlo approach. Our new analysis reveals higher SO_2_ oxidation rates than previously estimated, consistent with recent multiphase kinetics studies. Additionally, the potential relationship between changes in SO_2_ oxidation rates and sulfate formation pathways was elucidated by comparing these results to sulfate oxygen-17 anomalies. Our approach and findings offer a stringent assessment of how various sulfate formation pathways contribute to the overall SO_2_ oxidation rate in the planetary boundary layer and are therefore useful for evaluating the impacts of the atmospheric sulfur cycle on environmental health, public health, and climate.

## Graphical abstract

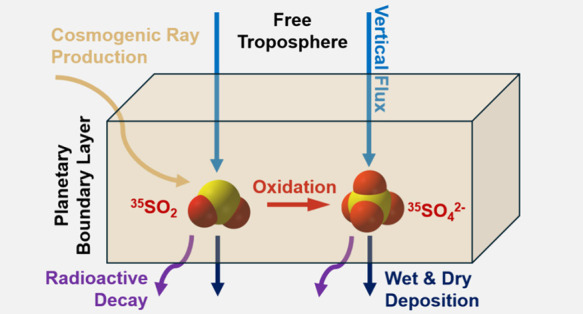

## 1 Introduction

Sulfate is a major chemical species in aerosols, playing an essential role in atmospheric chemistry, radiative forcing of climate, air quality, and public health.^1^ Sources of atmospheric primary sulfate include sea-salt aerosols and mineral dusts.^2^ The formation of secondary sulfate, a major part of sulfate aerosols, is predominantly controlled by the oxidation of sulfur dioxide (SO_2_) emitted anthropogenically (e.g., fossil fuel combustion) or naturally (e.g., volcanic activity and oxidation of biogenic dimethyl sulfide over oceans).^2^ Quantifying the oxidation rate of SO_2_ to sulfate aerosols is therefore critical for evaluating the budget of sulfate aerosols in the atmosphere and their effects on atmospheric, climate, and environmental systems.^1–3,19,22^

Conventional atmospheric chemistry models consider both gas- and aqueous-phase oxidation of SO_2_. The gas-phase oxidation of SO_2_ is primarily driven by its reaction with hydroxyl radicals (OH).^3^ Given the typical OH mixing ratio in the atmosphere (∼10^6^ cm^–3^), the estimated lifetime of SO_2_ with respect to gas-phase oxidation by OH is approximately 13 days. This lifetime is significantly longer than the overall atmospheric SO_2_ lifetime estimated by observations (ranging from hours to days),^4–7^ suggesting that aqueous-phase oxidation of SO_2_ must also be considered. Despite this, substantial sulfate concentration discrepancies between field observations and chemical models have been reported,^8^ indicating potential deficiencies in traditional sulfur chemistry frameworks. Recent laboratory-based kinetic studies have revealed that the reaction rate constants for various SO_2_ aqueous oxidation pathways in multiphase chemistry are considerably higher than previously understood from dilute bulk solution studies.^9–12^ These findings underscore the necessity of incorporating multiphase chemistry into atmospheric models. While field observations are crucial for guiding experimental studies and refining model predictions, quantifying SO_2_ oxidation rates in the complex ambient atmosphere and elucidating their relationships with various oxidation pathways remain challenging due to the diverse sulfur emission sources and associated chemical reactions. In particular, this quantification must account for emission inventories from multiple sulfur sources, which introduces great uncertainties.^2^ It was proposed that the calculation may be simplified by using radiosulfur (^35^S), as it originates from a single source.^2^

The radioactive isotope ^35^S, with a half-life of 87.4 days, is produced cosmogenically in the Earth’s atmosphere through the spallation of ^40^Ar by high-energy cosmic rays.^26^ Due to the higher flux of high-energy particles in the upper atmosphere and the elevated ^40^Ar mixing ratios in the lower atmosphere, the production rate of ^35^S is highest in the stratosphere, approximately 1–2 orders of magnitude greater than in the planetary boundary layer.^26^ Immediately following its production, ^35^S nuclides are oxidized to radiosulfur dioxide (^35^SO_2_) in the Earth’s oxidizing atmosphere within approximately 1 s and are then incorporated into the atmospheric sulfur cycle with chemical behavior akin to that of stable sulfur. ^35^SO_2_ is removed from the atmosphere through wet or dry deposition at the Earth’s surface or is further oxidized to radiosulfate (^35^SO_4_^2–^) via various oxidation pathways. ^35^SO_4_^2–^ is ultimately removed from the atmosphere via dry or wet deposition. Turekian and Tanaka were the first to utilize ^35^S to quantify sulfur deposition rates using box models,^13,14^ but relevant studies were extremely limited due to the analytical challenges of measuring ^35^S in the atmosphere.^25^ Advances in optimized ultralow-level liquid scintillation counting methods in the past decade^21,25,27^ have made ^35^S measurements for atmospheric samples feasible, particularly for both ^35^SO_2_ and ^35^SO_4_^2–^. Based on new ^35^SO_2_ and ^35^SO_4_^2–^ measurements,^24,28^ Lin et al.^2^ proposed that ^35^S is also effective in constraining SO_2_ oxidation rates, as it mimics the behavior of stable sulfur but is derived from a unique and quantifiable source, unlike the numerous sources of stable sulfur that are difficult to constrain. This approach relies on a box model that estimates ambient SO_2_ oxidation rates using measurements of ^35^SO_2_ and ^35^SO_4_^2–^, combined with known deposition data (Figure 1; see Section 2.2 for details). However, the study by Lin et al.^2^ remains preliminary due to unresolved uncertainties in model parameters, including deposition data. The uncertainty may be assessed using the Monte Carlo method, a technique that randomly generates a large number of values to address numerical problems associated with various sources of uncertainty.^29^

**Figure 1:**
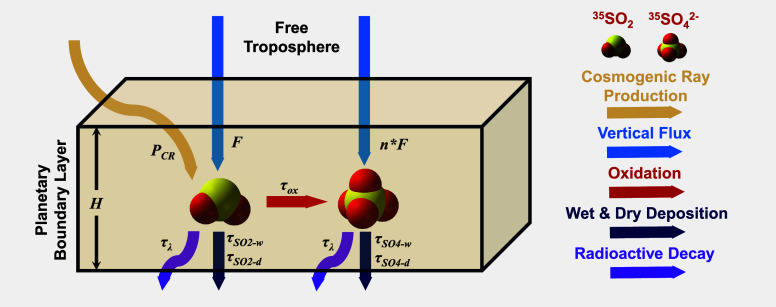
Schematic graph of a zero-dimensional ^35^S box model. Each parameter is indicated by colored arrows, with the legend provided on the right side. Refer to Section 2.2 for further details of parameters.

In this study, we present updated estimates of the SO_2_ oxidation rates in the planetary boundary layer based on combined measurements of ^35^SO_2_ and ^35^SO_4_^2–^. Our approach utilizes a comprehensive data set derived from nine global models for parametrization and incorporates the Monte Carlo method to thoroughly address uncertainties in parametrization. By leveraging these ^35^S-based calculation results alongside additional stable isotope signatures, we resolve the intrinsic relationship between elevated SO_2_ oxidation rates and variations in sulfate formation pathways.

## 2 Materials and Methods

### 2.1 Data Sources of Combined Measurements of ^35^SO_2_ and ^35^SO_4_^2–^

Existing measurements of ^35^SO_2_ and ^35^SO_4_^2–^ from the literature are compiled for analysis. We specifically focus on measurements obtained using optimized ultralow-level liquid scintillation counting methods,^25^ which include approximately three years of data collected in California and the Tibetan Plateau^24,28^ (Figure 2). Due to significant uncertainties associated with conventional analytical methods, earlier measurements from New England in the 1990s^13,14^ are excluded from this study.

**Figure 2:**
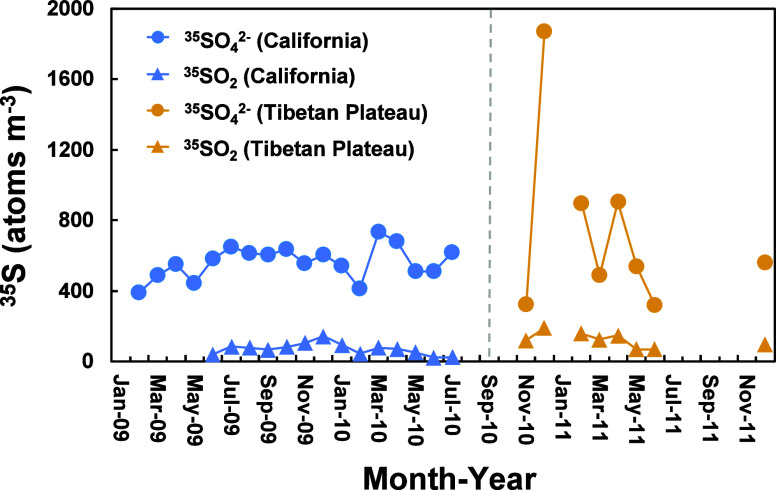
Time series of ^35^SO_2_ and ^35^SO_4_^2–^ in California^24^ and the Tibetan Plateau.^28^

California samples were collected at the Scripps Pier Shore Station (32.85°N, 117.28°W, 10 m above sea level),^24^ while Tibetan Plateau samples were collected at the Nam Co Monitoring and Research Station for Multisphere Interactions (30.77°N, 90.98°E, 4730 m above sea level).^28^ Air masses from both stations represent the Earth’s marine or terrestrial background atmosphere in the northern hemisphere with relatively minimal anthropogenic influences. The Scripps Pier Shore Station is predominantly influenced by natural marine emissions, such as biogenic sulfur-bearing species and sea-spray aerosols from the Pacific Ocean. However, anthropogenic emissions from the densely populated Los Angeles area and heavy ship traffic in the polluted marine boundary layer are also factors to consider.^30,31^ The Nam Co Station, situated in a pristine inland area, experiences a slight influence from long-range transport of air pollutants from South Asia.^32,33^ Data availability from the Tibetan Plateau is more limited compared to that from California due to operational challenges in the harsh environment. In these studies,^24,28^ total suspended particles (TSP) and SO_2_ samples were collected using high-volume aerosol samplers with an operational flow rate of ∼1 m^3^ min^–1^, with TSP collected on top and SO_2_ on KOH-impregnated backup filter papers. Each set of samples was collected continuously for 3–10 days.^20,23,24,28^ The samples were subsequently dissolved, purified, and converted to aqueous SO_4_^2–^ solutions, and the ^35^S activity was measured using an ultralow-level scintillation counter (Quantulus 1220). The analytical uncertainty for each measurement is less than 20% (one relative standard deviation).^27^ Detailed chemical analysis procedures and original data can be found in the referenced literature.^24,25,28^ Monthly average data were calculated, reported, and analyzed in this study. The ^35^SO_4_^2–^ concentrations for California TSP analyzed here are derived by summing the ^35^SO_4_^2–^ concentrations from both fine (<1.5 μm) and coarse (>1.5 μm) particulate fractions as reported in the literature.^24^

### 2.2 Radiosulfur Box Model

Using measured monthly ^35^SO_2_ and ^35^SO_4_^2–^ concentrations ([^35^SO_2_] and [^35^SO_4_^2–^], respectively; unit: atoms m^–3^), we can calculate the SO_2_ oxidation lifetimes (τ_ox_; unit: day) and downward vertical flux of ^35^SO_2_ from the free troposphere to the planetary boundary layer (*F;* unit: atoms m^–2^ day^–1^) employing a zero-dimensional radiosulfur box model with a box height of *H* (unit: m) (Figure 1). Following the methodology outlined by Lin et al.,^2^ we describe the time-dependent variation of ^35^SO_2_ and ^35^SO_4_^2–^ in the box model using the following equations:

d[SO235]dt=PCR+FH−[SO235]τox−[SO235]τSO2−w−[SO235]τSO2−d−[SO235]τλ 1

d[SO42−35]dt=nFH+[SO235]τox−[SO42−35]τSO4−w−[SO42−35]τSO4−d−[SO42−35]τλ 2

where *P*_CR_ is the production rate of ^35^SO_2_ in the boundary layer box (unit: atoms m^–3^ day^–1^), *n* the [^35^SO_4_^2–^]/[^35^SO_2_] ratio in the free troposphere, and τ_λ_ the decay lifetime of ^35^S [half-life/ln(2) = 126 days]. The parameters τ_SO2‑w_ and τ_SO2‑d_ represent the wet and dry removal lifetimes of SO_2_, respectively, while τ_SO4‑w_ and τ_SO4‑d_ represent the wet and dry removal lifetimes of SO_4_^2–^, respectively (unit: day).

The model is based on two key assumptions: (i) ^35^SO_2_ and ^35^SO_4_^2–^ concentrations are uniformly distributed vertically within the boundary layer, and (ii) horizontal fluxes of ^35^SO_2_ and ^35^SO_4_^2–^ are neglected. These assumptions are standard in box model analyses. As our sampling sites represent background conditions at a regional scale rather than local environments, we argue that these assumptions are physically reasonable, at least for our proof-of-concept study. In addition, given the rapid oxidation of cosmic-ray-produced ^35^S to ^35^SO_2_ (∼1 s),^34,35^ the production rate of ^35^SO_2_ in the planetary boundary layer is equivalent to the production rate of ^35^S nuclides. The production rate of ^35^SO_4_^2–^ is governed by the SO_2_-to-SO_4_^2–^ oxidation rate (Figure 1). Since the lifetimes of SO_2_ and SO_4_^2–^ in the planetary boundary layer (hours to days) are much shorter than one month, we apply a steady-state approximation in our calculation (i.e., d­[^35^SO_2_]/d*t* and d­[^35^SO_4_^2–^]/d*t* are set to zero). With appropriate parametrizations of known parameters (*n*, *P*_CR_ *, H,* τ_SO2‑w_, τ_SO2‑d_, τ_SO4‑w_, and τ_SO4‑d_), both the SO_2_ oxidation lifetime τ_ox_ and downward vertical flux *F* can be determined.

### 2.3 Parameterization of the Box Model and Monte Carlo Simulations

In this study, monthly ^35^SO_2_ and ^35^SO_4_^2–^ concentrations were obtained from data set described in Section 2.1. The monthly boundary layer heights (i.e., the model box height *H*) at the sampling sites were based on the ERA-20C reanalysis data set, averaging over the period from 2001 to 2010. Monthly wet and dry removal lifetimes of SO_2_ and SO_4_^2–^ at the sampling sites were derived from multiyear averages of deposition data from nine models in the Atmospheric Chemistry and Climate Model Intercomparison Project (ACCMIP)^36^ (Table 1). There are several uncertainties associated with the parametrizations described, as the ERA-20C reanalysis and ACCMIP model data (covering 2000–2010) do not perfectly align with the years of ^35^S measurements (2009–2011). To address uncertainties in interannual variabilities, we employed a Monte Carlo simulation method, where parameter values in each month were resampled multiple times within normal distribution ranges based on averages and one standard deviation calculated from multiyear data sets. For calculation using deposition data from model #8, uncertainties in deposition data were not considered due to the availability of only one year (2000) of data from this model (Table 1).

**Table 1:** Summary of Deposition Data Set Sources

model #	model	data set period
#1	GISS-E2-R	2000–2005
#2	NCAR-CAM3.5	2002–2009
#3	MIROC–CHEM	2000–2010
#4	CESM-CAM-Superfast	2000–2009
#5	HadGEM2	2000–2009
#6	NCAR-CAM5.1	2000–2009
#7	STOC-HadAM3	2000–2009
#8	CICERO-OsloCTM2	2000
#9	GFDL-AM3	2001–2010

For the production rate of ^35^SO_2_ (*P*_CR_), our earlier study^2^ followed the pilot calculation by Lal and Peters^26^ and estimated *P*_CR_ for California and the Tibetan Plateau to be approximately 1.6–1.9 and 42.3–52.9 atoms m^–3^ day^–1^, respectively, depending on the model box height *H*. According to the *H* values used in this study (based on 2001–2010 data from the ERA-20C reanalysis data set), the *P*_CR_ values for California and the Tibetan Plateau are estimated to be approximately 1.5 and 43 atoms m^–3^ day^–1^ (corresponding to *H* values of 440 ± 120 and 1060 ± 440 m; *n* = 120), respectively. The *P*_CR_ values calculated by Lal and Peters^26^ have not been updated for nearly 60 years, and their uncertainties remain poorly constrained. Our recent study of long-term ^35^S measurements suggested that the maximum *P*_CR_ value could be as much as double the minimum value across an 11-year solar cycle.^17^ To account for these uncertainties, we use uniform frequency distributions within the ranges of 1–2.5 and 27–68 atoms m^–3^ day^–1^ for California and the Tibetan Plateau, respectively, as the basis for Monte Carlo resampling. For the *n* value (i.e., the [^35^SO_4_^2–^]/[^35^SO_2_] ratio in the free troposphere), there are currently no direct measurements. Our earlier study examined three possible values (2, 2.3, and 3) inferred from previous observations and modeling.^2^ While uncertainties remain, the range of 2–3 is the most reasonable based on our current understanding. Similar to the treatment of resampling *P*_CR_ values, we used uniform frequency distributions within a range of 2–3 for *n* as the basis for Monte Carlo resampling to comprehensively account for its uncertainties.

Using the parametrization and Monte Carlo simulation strategy described above, we resampled parameters from each model to calculate τ_ox_ and *F* for 10,000 times at each site monthly. Since negative sulfur oxidation rates are not physically meaningful, only results with positive sulfur oxidation rates are presented and discussed in this study. The number of valid results for each month and site is summarized in Table S1. Totally, we obtained 896,720 valid results for California and 639,418 for the Tibetan Plateau from 1,400,000 and 800,000 resampling calculations, respectively. The relatively large fraction of unphysical oxidation rates in calculations using California data from model #8 (Table S1) is due to the model providing only one year (2000) of deposition data, which prevented us from conducting Monte Carlo resampling. Without adequate considerations of uncertainties, these parameters, derived from a year different from ^35^S measurements, resulted in a large fraction of physically meaningless numbers that we have excluded.

In this study, we focus solely on the interpretation of the calculated SO_2_ oxidation lifetime. Calculated *F* values (Figure S1) suggest that downward vertical fluxes from the free troposphere, relative to the cosmogenic production in the boundary layer, are the primary source of ^35^SO_2_ in both regions. This conclusion is physically reasonable, as ^35^SO_2_ is predominately produced in the upper atmosphere and subsequently removed through oxidation and deposition (Figure 1). Assuming that SO_2_ oxidation reactions in the planetary boundary layer follow pseudo-first-order kinetics, the sulfur oxidation rate *k* can be expressed as 1/τ_ox_ (unit: day^–1^).

## 3 Results and Discussion

### 3.1 Quantification of SO_2_ Oxidation Rates

The calculated oxidation rates of SO_2_ during the sampling period are presented in Figure 3, with averages of 2.0 day^–1^ for California and 1.6 day^–1^ for the Tibetan Plateau. The D’Agostino’s *K*-squared test indicates that the calculated values are not normally distributed at a significance level of 0.05. Therefore, we also examined the median values, which are 1.2 day^–1^ for California and 0.9 day^–1^ for the Tibetan Plateau. These results are generally higher than our previous estimates, which were based on deposition data sets from only two models (#2 and #6 in Table 1) and only several hundred calculations that inadequately addressed uncertainties.^2^ Additionally, we note that the variability of our results, as indicated by the interquartile range (IQR; the difference between the 75th and 25th percentiles of the data), is greater than our previous estimates.^2^ This increase in the variability reflects a more robust consideration of uncertainties in this study. Early experimental and model studies^37–48^ attempted to qualify the SO_2_ oxidation rates under ambient conditions, reporting first-order oxidation rates ranging from 0.3 to 6.1 day^–1^. Although these studies do not specifically focus on the two study regions examined in our work, our results are generally consistent with theirs.

**Figure 3:**
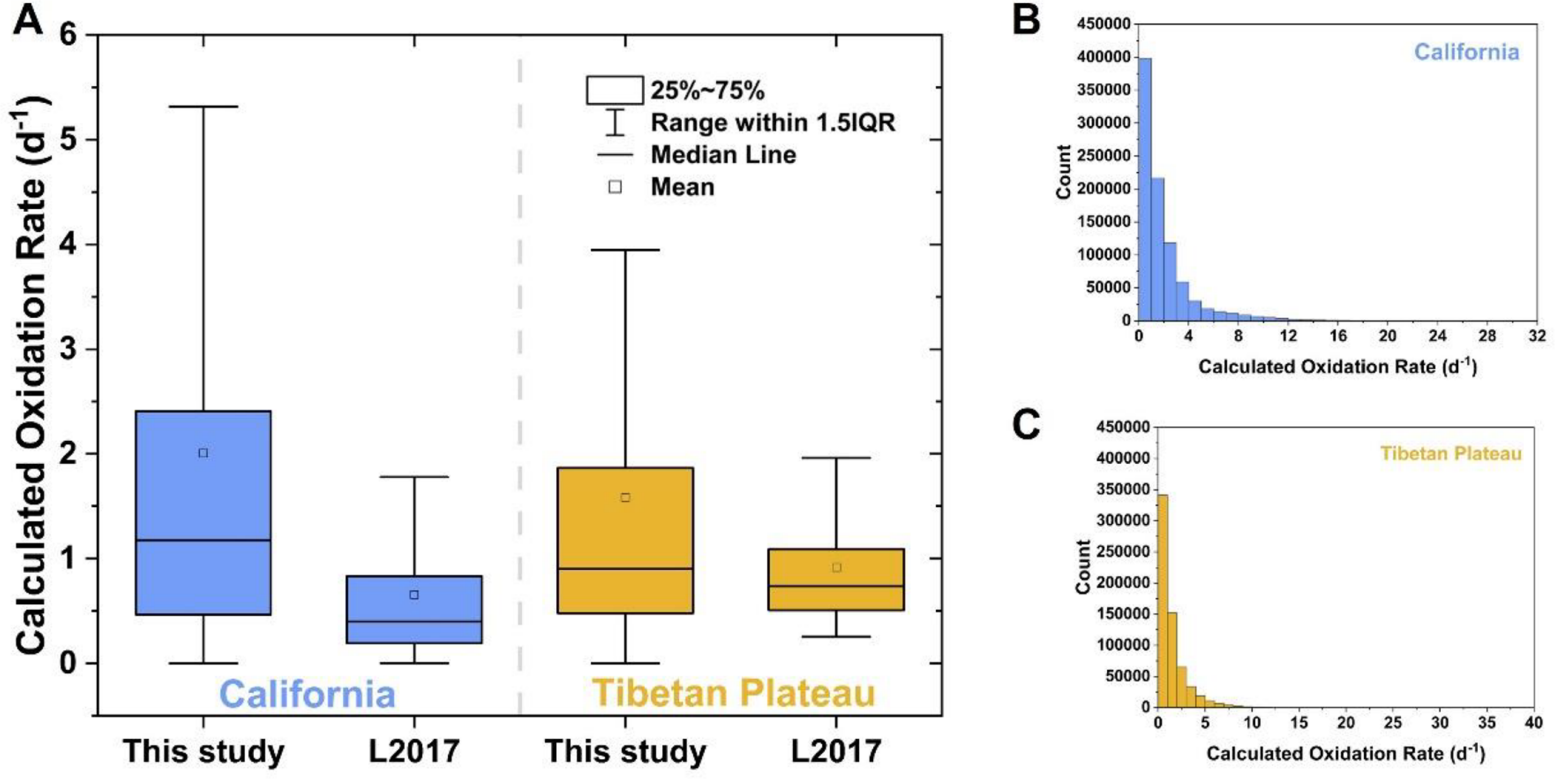
(A) Box-and-whisker plot of calculated SO_2_ oxidation rates in this study and Lin et al.^2^ (L2017); 1.5IQR stands for 1.5 times the interquartile range. (B) Histograms depicting the distribution of calculated SO_2_ oxidation rates for California. (C) Same as (B) but for the Tibetan Plateau.

Figure 4 illustrates the variability in SO_2_ oxidation rates estimated from Monte Carlo simulations using different deposition data sets from various models. For California, when considering only the deposition data from the two models used in our previous work^2^ (#2 and #6 in Table 1), the averaged SO_2_ oxidation rates are comparable to those previous estimates (Figures 3 and 4). However, calculations using deposition data from models #1, #5, and #7 yield higher SO_2_ oxidation rates. Examination of deposition data from all models listed in Table 1 reveals that the overall sulfate deposition velocities (including both dry and wet deposition) at the California sampling site (Scripps) are higher for models #1, #5, and #7 (>0.5 cm s^–1^) compared to others (0.1–0.3 cm s^–1^). The faster sulfate removal rates (i.e., shorter removal lifetimes) necessitate higher SO_2_ oxidation rates to match the observed ^35^SO_4_^2–^ concentrations under the steady-state assumption (Figure 1). This interpretation is corroborated by calculation results for the Tibetan Plateau, where the fastest SO_2_ oxidation rates are found using deposition data of models #3 and #5 (Figure 4), which also show the highest sulfate deposition velocities at the Tibetan Plateau sampling site (Nam Co). These results underscore the dependence of ^35^S-based SO_2_ oxidation rate estimations on the input of sulfur deposition data. The variability in sulfur deposition data among these ACCMIP models can be attributed to different model configurations.^36^ Despite these variations, the ACCMIP multimodel ensemble has been shown to be robust in quantifying sulfur deposition on a regional scale when compared to measurements.^49^ Consequently, using multimodel calculations is a common practice to enhance the output performance, as it comprehensively accounts for uncertainties and discrepancies.^36,49,50^ We therefore refrained from arbitrarily excluding any data from the nine different models in ACCMIP, as we lack an experimental basis to justify such exclusions.

**Figure 4:**
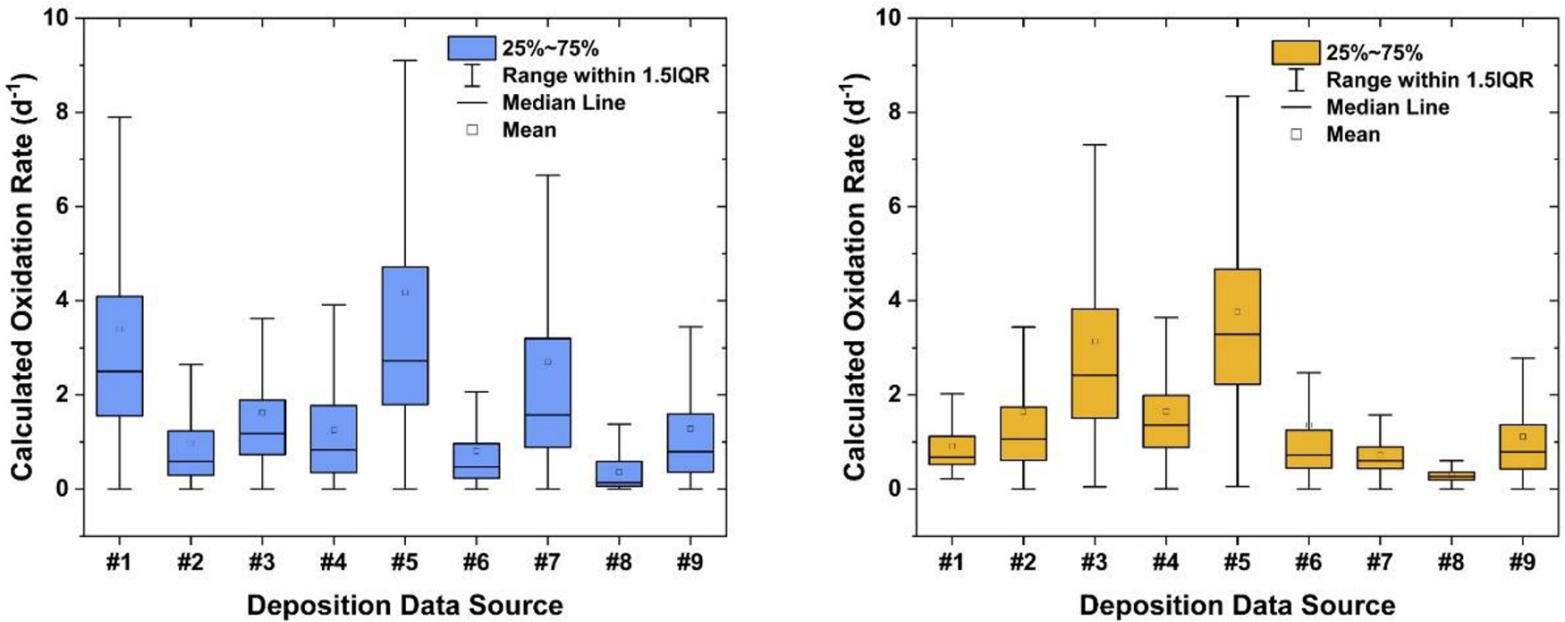
Oxidation rates calculated by the zero-dimensional ^35^S box model using various deposition data sets from the ACCMIP multimodel ensemble. 1.5IQR stands for 1.5 times the interquartile range. Left panel: California; right panel: Tibetan Plateau.

Despite considering deposition data sets from a multimodel ensemble to provide a comprehensive constraint on sulfur deposition parametrization, we note that the parameters *n* (the [^35^SO_4_^2–^]/[^35^SO_2_] ratio in the free troposphere) and *P*_CR_ (the production rate of ^35^S or ^35^SO_2_ in the boundary layer box) remain poorly constrained due to limited studies on these parameters. We further evaluate the sensitivity of calculated SO_2_ oxidation rates to the parametrization of *n* and *P*_CR_. Figure S2 demonstrates that the calculated SO_2_ oxidation rates at both sites are relatively insensitive to variations in *n* and *P*_CR_ within the Monte Carlo resampling ranges described in Section 2.3, highlighting the predominant influence of sulfur deposition parametrizations in determining SO_2_ oxidation rates using our ^35^S measurements and the Monte Carlo approach.

Overall, by utilizing nine different models from ACCMIP to comprehensively constrain deposition parameters and demonstrating the insensitivity of other poorly constrained parameters, we argue that the newly estimated SO_2_ oxidation rates from this study provide a more reasonable representation of natural conditions compared to our previous simple estimations.^2^ A key finding of this new calculation is the identification of a fast SO_2_ oxidation rate (Figure 3). Indeed, recent experimental studies have revealed that SO_2_ oxidation rates were previously underestimated.^9–12,51,52^ For instance, compared to traditional studies that only consider kinetics in diluted bulk solutions, the sulfate formation rates of aqueous oxidation pathways involving dissolved SO_2_ and oxidants such as hydrogen peroxide (H_2_O_2_), ozone (O_3_), and molecular oxygen (O_2_) (catalyzed by transition-metal ions) are dramatically faster (by 1–2 orders of magnitude) in multiphase reactions on the surface and in the bulk of aerosol particles, where additional factors like ionic strength must be considered.^10–12^ Furthermore, recent observational, kinetic, and dynamic studies have highlighted and elaborated on the role of nitrogen dioxide (NO_2_) in rapidly oxidizing SO_2_ in the aqueous phase.^9,51–54^ Although gas-phase oxidation of SO_2_ by OH was traditionally considered slow, recent research indicates that this sulfate formation pathway might be faster than previously thought in stratospheric intrusions, where OH mixing ratios are enhanced by interactions between O_3_-rich stratospheric air and H_2_O-rich tropospheric air.^55^ These new findings support the faster SO_2_ oxidation rates calculated in this study, as being kinetically reasonable.

### 3.2 Potential Relationships between SO_2_ Oxidation Rates and Sulfate Formation Pathways

In this section, we analyze the seasonal variability in SO_2_ oxidation rates derived from combined ^35^S measurements in SO_2_ and sulfate aerosols. We investigate major mechanisms potentially responsible for elevated SO_2_ oxidation rates in specific months by examining the relationship of SO_2_ oxidation rates with a unique isotopic tracer (the oxygen-17 anomaly)^56^ in the same sulfate aerosol samples. The oxygen-17 anomaly, expressed as Δ^17^O = δ^17^O – 0.52 ×δ^18^O, provides a distinctive isotopic signature useful for quantifying sulfate formation pathways.^56–58^ The δ^17^O and δ^18^O values are reported in delta notation (‰), calculated as δ^17^O = 1000‰×(^17^ *R*_sample_/^17^ *R*_VSMOW_ – 1) and δ^18^O = 1000‰ × (^18^ *R*_sample_/^18^ *R*_VSMOW_ – 1), where ^17^ *R* and ^18^ *R* represent the ratios of ^17^O/^16^O and ^18^O/^16^O, respectively, relative to the Vienna Standard Mean Ocean Water (VSMOW). Nonzero Δ^17^O values are “anomalous” because most oxygen-containing molecules on Earth typically exhibit Δ^17^O values close to 0‰ due to the mass-dependent fractionation rule (δ^17^O/δ^18^O ≈ 0.52) in stable isotope geochemistry.^56,59–61^ For example, Δ^17^O values for primary sulfate aerosols, which directly originate from the Earth’s surface (e.g., dust and sea-salts) or rapidly form during emissions such as volcanic eruptions and combustion, are approximately 0‰.^30,62,63^ Similarly, Δ^17^O values of SO_2_, water, and OH radical are close to 0‰.^56,57,64^ In contrast, Δ^17^O values of H_2_O_2_ and O_3_ are around 1.6 and 26‰, respectively, and these distinctive isotope signatures are transferred to sulfate aerosols during SO_2_ oxidation by these oxidants.^56–58,65,66^ Since the Δ^17^O values of sulfate aerosols remain unchanged after their formation, the Δ^17^O values inherited from oxidants are used to quantify the contributions of different SO_2_ oxidation pathways.^56^

Figure 5 displays the monthly SO_2_ oxidation rates calculated by using the Monte Carlo approach. For California, based on 14 months of ^35^S measurement data (June 2009–July 2010), the results reveal a bimodal seasonal pattern of SO_2_ oxidation rates, with increases observed at summer and winter. Notably, the peak observed during winter was more pronounced than what our previous simple calculations indicated.^2^ For the Tibetan Plateau, a clear seasonal pattern of SO_2_ oxidation rates could not be determined due to the limited 8-month data set. However, the data indicate that SO_2_ oxidation rates at our sampling site were generally higher in May, June, and December compared with February, March, April, and November. This monthly variability aligns generally with our previous estimates,^2^ although the magnitudes of the variation differ slightly. Interestingly, previous sulfate triple oxygen isotope measurements in California and the Tibetan Plateau also show relatively high Δ^17^O values in these seasons.^18,23,67^

**Figure 5:**
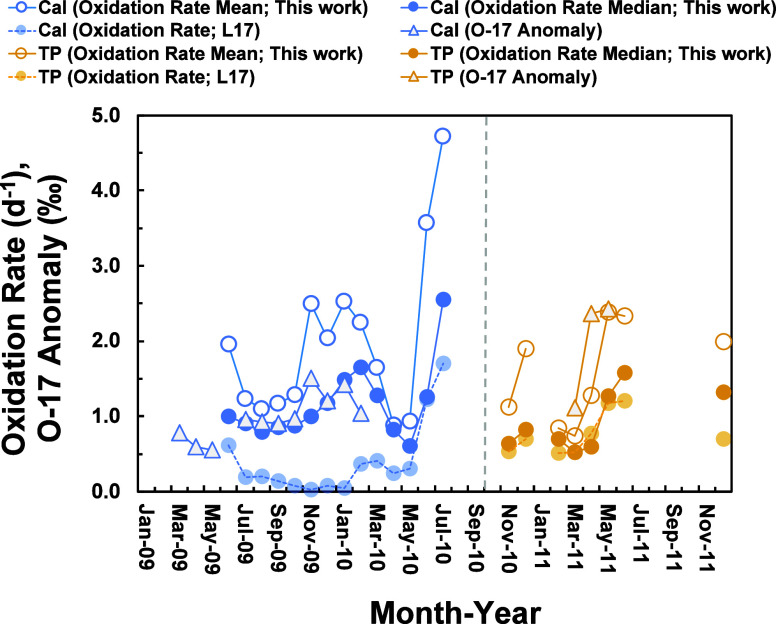
Time series of calculated SO_2_ oxidation rates and measured sulfate Δ^17^O values (oxygen-17 anomaly)^18,23^ in California (Cal) and the Tibetan Plateau (TP). L17 represents calculated SO_2_ oxidation rates in our previous works.^2^

Both the mean and median values of calculated oxidation rates in California exhibit an increase from late autumn to winter (November–February), though their variations differ (Figure 5). As discussed in Section 3.1, the normality assumption for the calculated results is not satisfied (Figure 3), suggesting that the median may be a more appropriate metric for investigation. Nevertheless, it is interesting to note that averaged SO_2_ oxidation rates in California during November–February appear to covary with the sulfate Δ^17^O values from the same samples^23^ (Figure 5). Since faster secondary sulfate production in the ambient atmosphere is typically associated with changes in environmental conditions that lead to different sulfate production mechanisms,^54^ the potential relationship between SO_2_ oxidation rates and sulfate Δ^17^O values may provide observational evidence for these changes. During this season, the sampling site at Scripps, located in coastal Southern California, is frequently affected by dry, hot, and strong winds descending from the inland desert, known as Santa Ana winds.^68^ Previous studies speculated that the elevated sulfate Δ^17^O values during this period were due to an increased contribution of sulfate aerosols from the free troposphere, which have higher Δ^17^O values, to the planetary boundary layer.^23^ However, direct evidence showing higher sulfate Δ^17^O values in the free troposphere has not been provided. In East Asia, sulfate Δ^17^O values in the free troposphere (∼2000 m above sea level) are identical to those in the boundary layer.^15^ Alternatively, the increased role of aqueous oxidation pathways involving H_2_O_2_ and O_3_ in sulfate formation during this season could explain the observation of increasing Δ^17^O values (from ∼1 to 1.5‰) (Figure 5). Both pathways lead to higher sulfate Δ^17^O values (∼0.8‰ for H_2_O_2_ and >6.5‰ for O_3_) than others.^56^ However, previous studies suggested that the relative contribution of these reactions, particularly the O_3_ oxidation pathway that requires a high pH (∼5.6) to dominate over the H_2_O_2_ pathway, did not increase in the boundary layer because an increased loading of alkaline mineral dusts was not observed, a speculation based on calcium concentrations.^23^ A recent kinetic study^11^ has shown that considering the ionic strength in multiphase chemistry, the aqueous oxidation rate of SO_2_ by O_3_ is more than 10 times faster than previously thought and can dominate over the H_2_O_2_ pathway even at low pH (∼4). Therefore, the elevated O_3_ mixing ratios during Santa Ana winds,^16^ which are frequently observed, likely increase both SO_2_ oxidation rates and sulfate Δ^17^O values, consistent with our observations (Figure 5). Moreover, November–February is the wet season in Southern California,^69,70^ and other SO_2_ multiphase oxidation pathways, such as H_2_O_2_ oxidation,^10^ are expected to play a significant role in increasing SO_2_ oxidation rates and sulfate Δ^17^O values when Santa Ana winds are not prevalent.

The fastest SO_2_ oxidation rates observed in this study in California occurred in June and July 2010 (Figure 5), but the sulfate Δ^17^O was not measured during this period. Nevertheless, sulfate Δ^17^O values appear relatively low in the summer based on measurements from 2009 (Figure 5). As discussed previously,^2^ SO_2_ aqueous oxidation by hypohalous acids (HOX) may significantly enhance the SO_2_ oxidation rates. This HOX oxidation pathway leads to sulfate aerosols with near-zero Δ^17^O values and has been proposed to be important (accounting for 33–50% of total sulfate formation) in the marine boundary layer during spring and summer.^71^ The role of HOX oxidation in coastal Southern California was also noted in a global three-dimensional chemical transport model study.^72^ It is, therefore, plausible that the rapid summer SO_2_ oxidation rate in coastal Southern California may be attributed to an enhanced role of HOX oxidation during the summer. This hypothesis could be tested through simultaneous measurements of ^35^SO_2_, ^35^SO_4_^2–^, and sulfate Δ^17^O in future studies.

Although both ^35^S and Δ^17^O measurements are limited in the Tibetan Plateau,^18^ existing data reveal a covariation between calculated SO_2_ oxidation rates and sulfate Δ^17^O values similar to that observed in California (Figure 5). The elevated Δ^17^O values highlight the significant role of O_3_ oxidation that would produce sulfates with high Δ^17^O values, particularly in light of the high pH levels (∼6) in fog, cloudwater, rain, and snow in the Tibetan Plateau region.^67^ In this high pH environment, O_3_ oxidation rates are notably faster than those of other oxidation pathways.^64^ At Nam Co, the highest monthly O_3_ mixing ratio is typically observed in May.^73^ The elevated O_3_ levels during this season may enhance sulfate aerosol formation rates through O_3_ oxidation, potentially explaining why both SO_2_ oxidation rates and sulfate Δ^17^O values peak concurrently in May (Figure 5). Recent sulfate triple oxygen isotope measurements at the Mt. Everest region also show relatively high Δ^17^O values compared to most low altitude sites,^67^ although no distinct May peak was observed. Interestingly, global three-dimensional atmospheric chemical transport model simulations for 2013 indicate a larger contribution of O_3_ oxidation in May. These results possibly reflect interannual variability.

Overall, we observed a covariation between SO_2_ oxidation rates calculated from this work and previously measured sulfate Δ^17^O values from both California and the Tibetan Plateau. These preliminary results suggest that our approach has the potential to identify the intrinsic link between the SO_2_ oxidation rates and pathways. In the ensuing section, we discuss future directions aimed at addressing the limitations of the current research to provide more accurate quantification of ambient SO_2_ oxidation rates and their relationship with sulfate formation pathways.

### 3.3 Future Directions

Our study provides a proof-of-concept approximation for calculating SO_2_ oxidation rates using simultaneous measurements of ^35^SO_2_ and ^35^SO_4_^2–^ along with well-founded assumptions that comprehensively consider uncertainties. We acknowledge that chemistry, transport, and deposition processes in the atmosphere are far more intricate than the simplified treatment presented in our zero-dimensional box model in which detailed chemical reactions were not incorporated. Future studies could be strategically designed to fully resolve SO_2_ oxidation rates and their relationship with sulfate formation pathways through the use of ^35^SO_2_, ^35^SO_4_^2–^, and sulfate Δ^17^O measurements. For instance, recent advancements in high-sensitivity analytical methods for radiosulfur and triple oxygen isotopes^21,27,74^ may enable high-temporal-resolution (daily or hourly) measurements to investigate rapid changes in SO_2_ oxidation mechanisms and rates. With direct measurements or improved parametrizations of ^35^SO_2_/^35^SO_4_^2–^ ratios in the free troposphere and deposition rates in the boundary layer, the uncertainties highlighted in this study could be significantly reduced. Such measurements will enhance our estimates and allow a more thorough assessment of how accurately the calculated oxidation rates reflect reality.

Future studies should employ sophisticated models that account for all relevant reactions to fully simulate sulfur chemistry in the planetary boundary layer. Particularly, the global chemical transport model GEOS-Chem has been employed to simulate sulfate Δ^17^O values, enabling the quantification of sulfate production mechanisms by comparing measured values.^67,75^ By integrating state-of-the-art cosmogenic isotope production rates, GEOS-Chem can also model cosmogenic radioberyllium (^7^Be and ^10^Be) to investigate atmospheric transport and deposition processes against field-based measurements.^76^ Given that radiosulfur can trace atmospheric transport, deposition, and sulfur oxidation rates, updating the ^35^S production rate and incorporating this radionuclide tracer into global chemical transport models are imperative. The combined isotope approach demonstrated in this study will provide unique and crucial observational constraints that can improve global chemical transport model predictions regarding the SO_2_ oxidation rates and pathways.

## 4 Conclusions

In this work, we quantified SO_2_ oxidation rates in the planetary boundary layer using a box model that incorporates simultaneous measurements of ^35^SO_2_ and ^35^SO_4_^2–^ alongside Monte Carlo simulations. The average (median) of calculated SO_2_ oxidation rates is 2.0 (1.2) day^–1^ for California and 1.6 (0.9) day^–1^ for the Tibetan Plateau. These results are higher than previously estimated, which aligns with recent studies suggesting that SO_2_ aqueous oxidation on aerosol surfaces occurs more rapidly than previously considered in cloudwater bulk solutions. By comparing these rates with Δ^17^O measurements in the same samples, we found a covariation between these two parameters, which may link the seasonal variation in SO_2_ oxidation rates to changes in the sulfate oxidation pathways. This comprehensive isotope approach provides a stringent assessment of how various SO_2_ oxidation pathways contribute to the overall sulfate formation rate in the planetary boundary layer.

## Supplementary Material



## Notes

Number of valid results in Monte Carlo simulations (Table S1), box-and-whisker plot and histograms of calculated *F*/*H* (Figure S1), and scatter plots illustrating the sensitivity relationship between calculated SO_2_ oxidation rates and control parameters *n* and *P*_CR_ (Figures S2) (PDF)
