# Supplementary material for: Isotopic Constraints on SO2 Oxidation Rates and Their Potential Relationship with Sulfate Formation Pathways in the Planetary Boundary Layer
Source: ACS Environ Au. 2024 Dec 19;5(3):267–76. doi: 10.1021/acsenvironau.4c00070 (PMC12100549; doi:10.1021/acsenvironau.4c00070)
Supplement: Supplementary file 1 [file vg4c00070_si_001.pdf]

Supporting Information for

Isotopic constraints on SO_2 oxidation rates and their potential relationship with sulfate formation pathways in the planetary boundary layer

Zhengwen Niu[1,2], Mang Lin[1]*

1. State Key Laboratory of Isotope Geochemistry, Guangzhou Institute of Geochemistry, Chinese Academy of Sciences, Guangzhou, 510640, China
2. College of Earth and Planetary Sciences, University of Chinese Academy of Sciences, Beijing, 100039, China

* Corresponding Author: linm@gig.ac.cn

This PDF file includes 4 pages (S1-S4), 1 table (Table S1), and 2 figures (Figures S1 and S2).

Table S1. Number of valid results in Monte Carlo simulations.

	Deposition Data Source[a]									
	#1	#2	#3	#4	#5	#6	#7	#8	#9	Overall
California (Scripps)										
JUN 09	6604	6672	6913	7021	8783	5528	10000	10000	6528	68049
JUL 09	7295	6929	7469	4158	9787	8162	9968	1956	5014	60738
AUG 09	8098	6916	7591	3164	8501	6878	9937	2200	4140	57425
SEP 09	9783	5023	8629	5082	7733	5766	9584	6112	6844	64556
OCT 09	9365	7768	7534	5281	8257	4776	7563	4089	7127	61760
NOV 09	8963	4163	6614	5090	6934	3256	3762	0	8680	47462
DEC 09	9036	4224	7132	4374	8359	2425	6122	0	7096	48768
JAN 10	9531	6007	8933	6388	8778	4091	4930	9	7990	56657
FEB 10	9226	7550	7897	7163	7937	6937	9109	10000	9043	74862
MAR 10	9949	9152	9185	6213	9562	7475	7919	9050	8401	76906
APR 10	9845	5906	8696	7572	7367	6786	9423	9073	7906	72574
MAY 10	5798	5631	7990	7469	7588	5345	9763	9294	5642	64520
JUN 10	6586	6791	6935	7129	8802	5453	9997	10000	6525	68218
JUL 10	7389	7861	7589	7415	9769	8540	9985	10000	5677	74225
Total	117468	90593	109107	83519	118157	81418	118062	81783	96613	896720
Tibetan Plateau (Nam Co)										
NOV 10	9921	9095	9904	9647	6628	7145	7518	9633	9341	78832
DEC 10	9037	7210	8271	6814	7898	7004	9898	10000	9713	75845
FEB 11	9538	8906	7919	6309	7795	8860	9815	10000	9273	78415
MAR 11	9766	9094	9132	6049	8183	9099	9838	10000	7894	79055
APR 11	9222	8634	9615	7695	8836	9164	9969	10000	7517	80652
MAY 11	9969	8964	9761	9731	9985	9312	9647	10000	9949	87318
JUN 11	9870	8407	9883	9103	9997	7341	9119	10000	9988	83708
DEC 11	9001	7169	8277	6771	7879	6969	9892	9949	9686	75593
Total	76324	67479	72762	62119	67201	64894	75696	79582	73361	639418

a. See Table 1 in the main text for details of nine models.

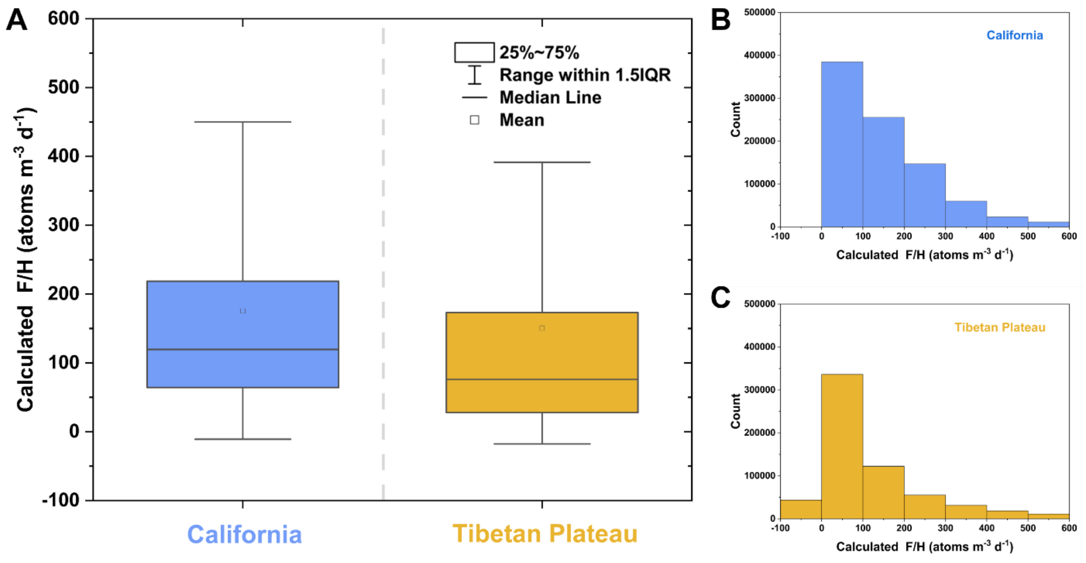

Figure S1. (a) Box-and-whisker plot of calculated *F/H* in this study; 1.5IQR stands for 1.5 times the interquartile range. (b) Histograms depicting the distribution of calculated *F/H* for California. (c) Same as Figure S1b but for the Tibetan Plateau.

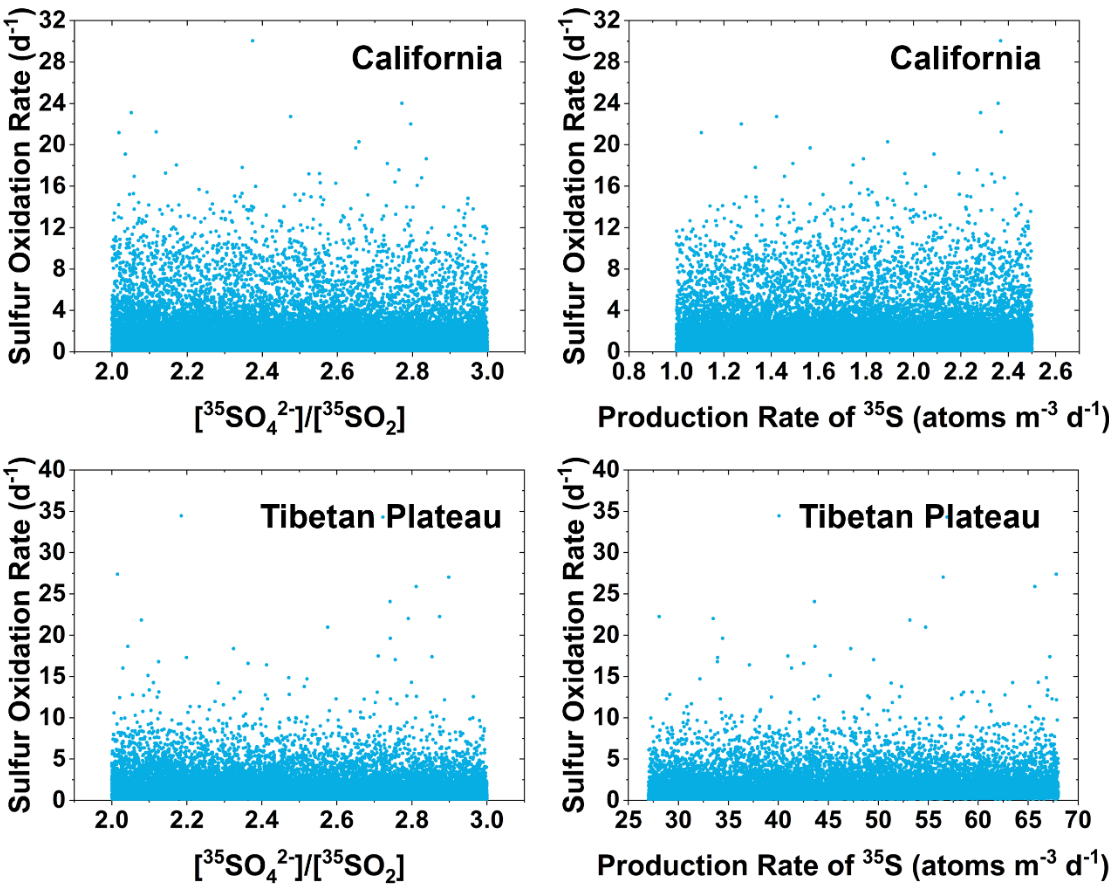

Figure S2. Scatter plots illustrating the relationship between calculated SO_2 oxidation rates and control parameters: (left) the [$^{35}SO_4^{2-}$]/[$^{35}SO_2$] ratio in the free troposphere, and (right) the production rate of ^{35}S in the planetary boundary layer.
